# Extreme Hypofractionation with SBRT in Localized Prostate Cancer

**DOI:** 10.3390/curroncol28040257

**Published:** 2021-08-03

**Authors:** Maria Antonia Gómez-Aparicio, Jeannette Valero, Begoña Caballero, Rafael García, Ovidio Hernando-Requejo, Ángel Montero, Alfonso Gómez-Iturriaga, Thomas Zilli, Piet Ost, Fernando López-Campos, Felipe Couñago

**Affiliations:** 1 Department of Radiation Oncology, Hospital Universitario de Toledo, 45007 Toledo, Spain; mariang.aparicio@gmail.com; 2 Department of Radiation Oncology, Hospital Universitario HM Sanchinarro, 28050 Madrid, Spain; jvalero@hmhospitales.com (J.V.); ohernando@hmhospitales.com (O.H.-R.); angel.monteroluis@gmail.com (Á.M.); 3 Department of Radiation Oncology, Hospital Universitario de Fuenlabrada, 28942 Fuenlabrada, Spain; begocaba@gmail.com; 4 Department of Radiation Oncology, Hospital Ruber Internacional, 28034 Madrid, Spain; rafa_gg2001@yahoo.es; 5 Department of Radiation Oncology, Cruces University Hospital, 48903 Barakaldo, Spain; agomeziturriaga@gmail.com; 6 Department of Radiation Oncology, Geneva University Hospital, 1205 Geneva, Switzerland; thomas.zilli@hcuge.ch; 7 Department of Radiation Oncology, Ghent University Hospital, 9000 Ghent, Belgium; Piet.Ost@UGent.be; 8 Department of Radiation Oncology, Hospital Universitario Ramón y Cajal, 28034 Madrid, Spain; 9 Department of Radiation Oncology, Hospital Universitario Quirónsalud, 28223 Madrid, Spain; fcounago@gmail.com; 10 Department of Radiation Oncology, Hospital La Luz, 28003 Madrid, Spain; 11 Clinical Department, Faculty of Biomedicine, Universidad Europea, 28670 Madrid, Spain

**Keywords:** extreme hypofractionation, hypofractionation, prostate cancer, stereotactic body radiation therapy (SBRT)

## Abstract

Prostate cancer is the most commonly diagnosed cancer among men around the world. Radiotherapy is a standard of care treatment option for men with localized prostate cancer. Over the years, radiation delivery modalities have contributed to increased precision of treatment, employing radiobiological insights to shorten the overall treatment time, improving the control of the disease without increasing toxicities. Stereotactic body radiation therapy (SBRT) represents an extreme form of hypofractionated radiotherapy in which treatment is usually delivered in 1–5 fractions. This review assesses the main efficacy and toxicity data of SBRT in non-metastatic prostate cancer and discusses the potential to implement this scheme in routine clinical practice.

## 1. Introduction

External beam radiation therapy (EBRT) is considered one of the standard treatments for organ-confined prostate cancer (PCa), with cure rates similar to those of radical prostatectomy. Hypofractionation uses a higher dose-per-fraction of radiation, which reduces the number of fractions and the total duration of treatment, allowing greater comfort for the patient and lower costs, in addition to providing a therapeutic advantage in terms of tumor control and toxicity, as the α/β of PCa is lower than that of adjacent healthy tissues [1]. In 2018, a group of experts from the American Societies of Radiation Oncology, Medical Oncology, and Urology (ASTRO/ASCO/AUA) concluded that there is sufficiently robust evidence to justify using moderate hypofractionation in PCa as common clinical practice [2], and a recent Cochrane review indicated that moderate PCa hypofractionation (with fractions up to 3.4 Gy) provides oncological outcomes in terms of overall survival (OS), disease-free survival (DFS), and metastasis-free survival (MFS) similar to conventional fractionation, without a significant increase in acute or late toxicity [3].

In addition, technical advances in the field of radiotherapy in recent years, such as intensity-modulated radiotherapy (IMRT), image-guided radiotherapy (IGRT), and stereotactic radiotherapy (SBRT), have enabled the progressive implementation of extreme hypofractionation (defined by fractions of at least six Gy) in various scenarios of localized PCa treatment. The use of SBRT in PCa has provided sufficient evidence in terms of tumor control results, quality of life reported by the patient, and low toxicity [4,5,6] to back its implementation in daily clinical practice. Moreover, the PCa working group of the German Society of Oncology (DEGRO) endorses the use of SBRT in the treatment of localized low- and intermediate-risk PCa, recommending its use in clinical trials in patients with the localized high-risk disease [7].

The recent publication of two randomized trials comparing the use of extreme hypofractionation versus conventional fractionation (HYPO-RT-PC [5], PACE-B trial [6]) has been crucial in supporting its use, although only the Scandinavian study (HYPO-RT-PC) reported results of long-term tumor and toxicity control. In 2020, a randomized systematic review and meta-analysis of phase III trials were published comparing SBRT with normofractionated and hypofractioned regimens. It concluded that the ultra-hypofractionated regimens obtained similar 5-year disease-free survival results, with late gastrointestinal and genitourinary toxicity of <15% and <21%, respectively, when compared to hypofractionated regimens and conventional radiotherapy [8].

Moreover, the COVID-19 pandemic has put great pressure on oncology services, making ultra-hypofractionated treatments even more attractive. Its shorter duration facilitates a reduction in the risk of infection of patients and staff. As a result, the international recommendations in response to COVID-19 for treating PCa indicate that schedules of 5–7 fractions should be used on the disease in those centers that have the appropriate technology [9].

At a time when there is growing interest in adopting extreme fractionation schedules in treating PCa in our usual practice, this review aims to capture the current evidence and recommendations for using it in different scenarios of PCa treatment.

## 2. SBRT in Low- and Intermediate-Risk Prostate Cancer

Extreme hypofractionation in low- and intermediate-risk PCa is currently considered a valid alternative to conventional normofractionated or moderate hypofractionated EBRT schedules.

The results of the SHARP trial, published by Madsen et al. [10] in 2007, confirmed what was happening in other pathologies: SBRT in PCa was feasible, with an acceptable toxicity profile. This was the first prospective study published, and it included 40 patients with low-risk PCa treated with 33.25 Gy in five fractions. With a median follow-up of 41 months, the biochemical relapse-free survival (bRFS) rate was 70% without signs of grade 3 or higher toxicity. Following the initial publication of phase I/II trials, numerous prospective studies assessing local control and tolerance of SBRT schedules have been published (Table 1). In 2018, Fuller et al. published a prospective series of 259 patients with low-risk and intermediate-risk PCa treated with 38 Gy in four fractions. With a median follow-up of five years, the biochemical control was 100% and 88.5% for the low-risk and intermediate-risk group, respectively. In terms of toxicity, 2.3% of patients developed grade ≥ 3 genitourinary toxicity, while no patients developed grade > 2 gastrointestinal toxicity [11]. This data is similar to that reported by Meier et al., who evaluated the tolerance and survival of 309 patients treated with 40 Gy in five fractions with a bRFS rate of 97.1% at five years with no evidence of grade ≥ 3 acute toxicity and 1.3% of grade 3 chronic genitourinary toxicity [12].

Elsewhere, other groups, such as Zelefsky et al., have published the results of a 5-year dose-escalation trial of 136 patients with localized low-risk and intermediate-risk PCa. The initial dose was 32.5 Gy in five fractions and scaled sequentially to 35 Gy, 37.5 Gy, and 40 Gy. No patients had grade ≥ 2 late gastrointestinal toxicity, and only one patient (0.7%) had grade 3 late genitourinary toxicity. At 12 and 24 months, the IPSS score in the group that received 40 Gy was higher than the score in the group that received 32.5 Gy, but no differences were observed at 36 months between the different cohorts [13]. The same group has just published the results of 257 prostate biopsies, performed two years after the end of SBRT to identify predictors for positive response [14]. The doses used were the same as in the previous trial, although it should be noted that the majority of patients, 68%, received 40 Gy in five fractions. <40 Gy doses were associated with positive biopsy results (*p* = 0.008). There was a significant correlation between a positive biopsy and the risk group, and the association was stronger in high-risk and unfavorable intermediate-risk than in low-risk (OR: 7.25, 95% CI: 2.69–19.55). Patients with a positive biopsy were at higher risk of developing a biochemical recurrence at five years (*p* < 0.001). In terms of quality of life, Alayed et al. carried out a comparative analysis of two prospective studies: 2START [15] and PATRIOT [16], without observing large differences, although the 2-fraction regimen was better tolerated in terms of gastrointestinal quality of life [17].

**Table 1:** Prospective studies of extreme hypofractionation in patients with localized low- and intermediate-risk PCa.

Reference	Risk Group	N Patients	RT Regimen (Gy)	Biochemical Failure-Free Survival	≥3 Acute Toxicity (%)	≥3 Chronic Toxicity (%)
[10]	Low-Risk	40	5 fr × 6.5	90% at 2 years	GU 2.5% GI 0%	GU 0% GI 0%
[18]	Low-Risk	45	5 fr × 7.5 5 fr × 7.25	92.7% at 5 years	GU 2% GI 0%	GU 1% GI 5%
[19]	Low-Risk	84	5 fr × 7	93% at 5 years	GU 1% GI 0%	GU 1% GI 1%
[20]	Low-Risk Intermediate-Risk	91	5 fr × 9 5 fr × 9.5 5 fr × 1	98.6% at 5 years	GU 0% GI 2%	GU 5,5% GI 7%
[11]	Low-Risk Intermediate-Risk	259	4 fr × 9.5	100% LR, 88.5% IR at 5 years	GU 1.1% GI 0%	GU 1.9% GI 0%
[12]	Intermediate-Risk	350	5 fr × 8	97.1% at 5 years	GU 0% GI 0%	GU 1.3% GI 0%
[13]	Low-Risk Intermediate-Risk	136	5 fr × 6.5 5 fr × 7 5 fr × 7.5 5 fr × 8	NR	GU 0% GI 0%	GU 0.7% GI 0%
[15]	Low-Risk Intermediate-Risk	30	2 fr × 13	96.7% at 5 years	GU 0% GI 0%	GU 3.3% GI 3.3%
[16]	Low-Risk Intermediate-Risk	152	5 fr × 8	95.7% at 5 years	NR	NR

Abbreviations: LR, low-risk; fr, fractions; GI, gastrointestinal; GU, genitourinary; NR, not reported; IR, intermediate-risk; RT, radiotherapy.

Furthermore, a prospective, multicenter phase II study has recently been published, evaluating toxicity after a year of SBRT treatment using three fractions in PCa of low- and favorable intermediate-risk. Fifty-nine patients were evaluated, and they were treated with 40 Gy in three fractions. Acute gastrointestinal toxicity was occasional, while 11.9% of patients experienced grade 2 acute genitourinary toxicity and 1.7% grade 3. However, no patient had persistent symptoms at 12 months post-treatment [21].

Finally, the results of the PROSINT trial have been published [22]: It was a prospective phase II study comparing 45 Gy in five fractions versus 24 Gy in a single fraction in 30 patients with intermediate-risk PCa. With a 48-month median follow-up, there were no significant differences in bRFS and acute toxicity between the two arms. It will be necessary to wait for the outcome of the PROSINT II, with 200 patients, and the ONE-SHOT trials to establish the efficacy and safety of a single fraction SBRT [23].

### 2.1. Reviews and Meta-Analyses of Non-Randomized Studies

There are multiple published prospective trials in which thousands of patients with extreme hypofractionation have been treated, with different doses, fractions, and techniques. The most relevant have been analyzed in four reviews or meta-analysis studies.

The first of these was published in 2013 [24], and it analyzed 1100 patients treated with CyberKnife in prospective phase II trials. The median dose used was 36.25 Gy (range 35–40 Gy) in five fractions. The percentage of patients included with localized low-risk, intermediate-risk, and high-risk PCa was 58%, 30%, and 11%, respectively. With a median follow-up of 36 months, the bRFS rate at five years was 93% in the whole cohort of patients, with no differences observed among delivered total doses, and no toxicity data reported. Kishan et al. analyzed data from 12 prospective phase II trials, with a total of 2142 patients with localized low-risk and intermediate-risk PCa. With a median follow-up of 6.9 years, biochemical recurrence was 4.9% at low-risk and 10.2% at intermediate-risk, with 0.6% grade ≥ 3 acute genitourinary and 0.09% gastrointestinal toxicity, and 2.1% grade ≥ 3 late genitourinary and 0.3% gastrointestinal toxicity, respectively [25]. Cushman et al. published the results of a systematic review and meta-analysis of 14 prospective studies, with a total of 2038 patients. Most patients were low-risk and intermediate-risk (51% and 37%, respectively). With a median follow-up of 3.1 years, the bRFS rate was 98%, with 1% ≥ 3 late gastrointestinal toxicity and 2% genitourinary toxicity [26].

The most recent meta-analysis and the most extensive review to date was conducted by Jackson et al. [4] in 2019. Six thousand, one hundred and sixteen patients were part of 36 prospective trials, and 92%, 72%, and 38% of the studies included low-risk, intermediate-risk, and high-risk patients, respectively. The median follow-up was 3.3 years, describing a five-year bRFS rate of 96.7% for low-risk and 92.1% for intermediate-risk patients, with less than 1% ≥ 3 acute toxicity, 2% ≥ 3 late genitourinary, and 1% gastrointestinal toxicity.

In 2019, Roy and Morgan published a review of the hypofractionated and ultra-hypofractionated studies in localized prostate cancer. They concluded that SBRT is an option in treating patients with low and intermediate-risk, even though the evidence remains less solid than that supporting the use of moderate hypofractionation in these groups of risk [27].

A review of studies conducted between 2001 and 2018 evaluating genitourinary, gastrointestinal, and sexual effects in patients treated with SBRT has recently been published [28], describing a relationship between dose received and genitourinary and gastrointestinal toxicity. However, there are few studies that examine the possible relationship between erectile dysfunction and dose to organs at risk (OAR) [29].

### 2.2. Phase III Randomized Studies

To date, only two randomized phase III studies have been published: HYPO-RT-PC [5] and PACE-B [6].

Pending the results of biochemical control and late toxicity of PACE-B, Widmark et al. establish the bases of what could be the standard of care in patients with localized PCa, considering certain features.

#### 2.2.1. HYPO-RT-PC

It was a randomized, multicenter, prospective phase III study comparing normofractionation and extreme hypofractionation in patients with intermediate- (89%) and high-risk (11%) PCa disease. One thousand, one hundred and eighty patients took part—591 were treated with 78 Gy in 39 fractions, and 589 with 42.7 Gy in seven fractions, in 2.5 weeks. Most of the patients were treated with 3D radiotherapy, something that seems obsolete in the IMRT era, without allowing the use of androgen deprivation and using 7 mm expansion to generate planning target volume (PTV). After a 5-year follow-up, the bRFS rate was 84% in both groups, with no differences in overall survival rates or acute and late gastrointestinal toxicity. In terms of genitourinary toxicity, a non-significant increase in grade ≥ 2 acute toxicity was observed in the extreme hypofractionated arm compared to the control arm (28% vs. 23%), with no differences between the two arms at the 5-year follow-up.

#### 2.2.2. PACE-B

It was a randomized, multicenter, phase III trial comparing standard fractionation and extreme hypofractionation with SBRT in localized low- and intermediate-risk PCa. Eight hundred and seventy-four patients were evaluated—441 were treated with 78 Gy in 39 fractions or 62 Gy in 20 fractions and 433 with 36.25 Gy in five fractions, on consecutive days or on alternate days. Acute toxicity data according to RTOG criteria did not show significant differences between the two arms in grade ≥ 2 gastrointestinal or genitourinary toxicity.

### 2.3. SBRT vs. Brachytherapy

There are no prospective studies comparing brachytherapy with SBRT. However, the results of retrospective studies in terms of biochemical control and toxicity do not show differences between the two treatment techniques, which would have to be confirmed by randomized studies [30,31].

## 3. SBRT in High-Risk Prostate Cancer

SBRT treatment in localized high-risk PCa is controversial. Most SBRT studies that include patients with high-risk PCa are observational. In the prospective studies, the number of high-risk patients is low, so there is insufficient evidence to support its routine use at this time outside clinical trials.

In 2018, González-Motta et al. [32] published a review of studies that include high-risk patients treated with extreme hypofractionation, evaluating 20 studies, of which 13 were SBRT in monotherapy, 5 were SBRT as a boost, and 2 were mixed studies. The doses used in the monotherapy studies range from 32–40 Gy in 4–5 fractions, and for the SBRT as a boost, the doses range from 10 Gy in two fractions to 21 Gy in 2–3 fractions. On the other hand, in a recent systematic review for high-risk PCa of 23 relevant studies, the authors concluded that SBRT with or without pelvic elective nodal irradiation could not be considered the standard of care in these patients, due to missing level 1 evidence. Treatment may be offered to selected patients at specialized centers with access to high-precision radiotherapy [33].

### 3.1. SBRT as Monotherapy

A prospective analysis of 344 SBRT-treated patients with a median follow-up of 4.1 years has recently been published, describing a bRFS rate of 81.7% and an MFS rate of 89.1% [34]. Katz et al. [35] provide the longest follow-up data for this subgroup, with a median follow-up of eight years. DFS was 65% when high-risk patients were included, with no differences in biochemical control observed between patients treated with 35 Gy or 36.25 Gy in five fractions. Likewise, HYPO-RT-PC [5] included 126 high-risk patients, of whom 62 (11% of the total) were treated with extreme hypofractionation, although such sample size is too small to extrapolate the results of the study to this subgroup.

### 3.2. SBRT as a Boost

The administration of combined ERT treatments, which additionally applied a boost with SBRT, has been analyzed in a lower percentage of patients. The follow-ups in published studies have been short [36,37].

The PROMETHEUS study, a prospective phase II, evaluated toxicity and tumor control in 135 patients, of whom 32 (24%) were patients with high-risk PCa, after administering 46 Gy in 23 fractions and a boost of 19–20 Gy in two fractions. With a median follow-up of two years, the bRFS rate was 98.6%, without describing grade ≥ 2 acute genitourinary or gastrointestinal toxicity, and 2% grade 3 late toxicity resolved within 20 months of completion [38].

Eade et al. published the results of a prospective phase I dose-escalation study, which compared a 46 Gy, 22 Gy, and 24 Gy boost in the prostate with 25 Gy, 27.5 Gy, and 30 Gy to the dominant lesion, respectively in two fractions, after administration of 46 Gy in 23 external radiotherapy fractions. With a median follow-up of two years, the three-year bRFS rate was 93.3%, without describing grade ≥ 2 toxicity in any of the groups analyzed [39].

Additionally, the HYPO-PROST trial is a randomized controlled trial comparing two different prostate boost schedules (30 Gy in 15 fractions vs. 15 Gy in two fractions) administered after 46 Gy in 23 fractions to prostate, seminal vesicles, and pelvic nodes. After a median follow-up of 60.1 months, bRFS at five years was 82.9% in the normofractionated regimen versus 78.2% in the ultra-hypofractionation group. No differences were observed in terms of late gastrointestinal or genitourinary toxicity between the two treatment regimens [40].

### 3.3. SBRT and Androgen Deprivation Therapy

The use of ADT concomitant with normofractionated radiotherapy and moderate hypofractionation is now well established, and randomized clinical trials have shown an improvement in survival with the addition of ADT to normo- and hypofractionated regimens in intermediate and high-risk patients [41,42,43].

Despite the high level of available evidence of increased overall survival with ADT addition to normal and hypofractionated radiotherapy in high-risk patients, its appearance in SBRT studies with this subgroup of patients is very heterogeneous, with its use in some series being omitted [32,33], and widespread omission of ADT in published studies of intermediate-risk patients [11,12].

Royce et al. examined the use of ADT in 7559 patients treated with SBRT, versus 133,825 treated with hypofractionation or conventional radiotherapy, using data obtained from the USA National Data Base. In the multivariate analysis, they showed that the use of ADT was significantly lower in patients who received SBRT in comparison with those who received conventional radiotherapy, or with moderate hypofractionation in all risk groups (*p* < 0.001) [44]. Recently, the results of the study carried out by van Dams et al. were published, which evaluated 344 high-risk prostate cancer patients treated with SBRT. 69% of them received ADT for an average of nine months. The use of ADT was associated with an increase in bRFS in the multivariate analysis (*p*-value: 0.009), although without any difference in metastasis-free survival. [34].

The only phase III trial of SBRT to date with published results that included high-risk patients did not allow the use of ADT [5]. The results of the ongoing PACE-C trial comparing hypofractionated radiation therapy plus ADT versus SBRT plus ADT in intermediate and high-risk patients could potentially be revealing in this regard.

At this time, we cannot establish the true role of ADT or its optimal duration in high-risk patients treated with SBRT, given the lack of sufficient levels of evidence for this subgroup of patients. Regarding the optimal sequencing to administer treatment with ADT in combination with SBRT in localized prostate cancer, there are no data in published studies in which ADT is used that indicate the optimal period to start ADT treatment [34,44], using as reference the timing established in studies employing normo- or moderate hypofractionated regimens [41,42,43].

### 3.4. SBRT and Prophylactic Nodal Irradiation

Prophylactic nodal irradiation with normofractionated radiotherapy in patients with high-risk PCa has shown an increase in bRFS and DFS rates without showing benefits in OS [45]. Three studies have analyzed prophylactic lymph node irradiation with SBRT to date, one of them prematurely halted because of high rates of genitourinary and gastrointestinal toxicity [46].

Alayed et al. compared two prospective phase II trials, with and without lymph node irradiation. The fractions used in each of them were 40 Gy in five fractions to prostate plus 30 Gy in five fractions to seminal vesicles vs. 40 Gy to prostate and 25 Gy to seminal vesicles and pelvis, in five fractions. The results for OS were similar in both trials (93.2% vs. 96.7%) with a biochemical failure rate at five years without and with lymph node irradiation of 14.6% and 0%, respectively.

No grade toxicity 3 to 4 late GU toxicity was reported in any trial. Only one patient had grade 3 late GI toxicity in lymph node irradiation group. Overall, patients receiving lymph node irradiation presented a higher rate of late gastrointestinal toxicity (grade 1 53% vs. 30%, grade 2 37% vs. 30% and grade 3 3% vs. 0%), (*p* = 0.009), with no differences in genitourinary toxicity (grade 1 37% vs. 30%, grade 2 57% vs. 60% and grade 3 0% in both groups), (*p* = 0.04) with and without lymph node irradiation, respectively [47]. Murthy et al. published a study evaluating toxicity in 68 patients with high-risk PCa treated with 35–37.5 Gy in five fractions to prostate and 25 Gy to pelvis in five fractions, with no grade ≥ 3 acute toxicities and with late genitourinary and gastrointestinal toxicity of 3% and 0%, respectively, which was similar to studies without lymph node irradiation [48].

## 4. SBRT on Prostate Bed

Radical prostatectomy remains a standard treatment for prostate adenocarcinoma. However, up to 33% of patients will experience a biochemical relapse at follow-up [49,50].

Postprostatectomy radiotherapy includes both early adjuvant radiotherapy after surgery (ART) and salvage radiotherapy in patients with a recurrence of abnormal PSA levels after surgery (biochemical relapse). To demonstrate the viability and safety of the use of SBRT in this clinical scenario, Repka et al. [51] conducted a theoretical feasibility study of SBRT after prostatectomy based on the NTCP (Normal Tissue Complication Probability) model, using patients who had previously been treated by conventional EBRT for biochemical recurrence after prostatectomy. Using the presimulation CT, RTOG recommendations were applied to define postprostatectomy volumes, and a dose of 30 Gy was prescribed to the PTV in five fractions, corresponding to an equivalent dose in 2 Gy fractions (EQD2) of 64.3 Gy, assuming an α/β value of 1.5. The NTCP model was applied to estimate the risk of late rectal and/or bladder toxicity. According to the NTCP model, the mean of grade ≥ 2 late rectal toxicity was estimated at 0.28% (±0.03%) and of late grade 2 toxicity on the bladder neck at 0.00013% (±0.000084%), while the calculated average for the exacerbation of late urinary symptoms was 4.81% (±0.52%). The conclusion by the authors, considering the limitations of the NTCP model, is that using SBRT after surgery seems feasible and may offer a safe, convenient treatment option for patients in both the adjuvant and salvage after biochemical failure. Table 2 shows a comparative analysis of equivalent doses in terms of EQD2 and biological effective dose (BED) with three different treatment schedules (normofractionation, moderate hypofractionation, and ultra-hypofractionation or SBRT).

In recent years, various studies have explored the possibility of administering SBRT for treating the prostate bed (Table 3). Sampath et al. performed a postprostatectomy SBRT dose-escalation study up to 45 Gy in five fractions. Although 45 Gy in five fractions was safe for prostate bed SBRT, it did not improve clinical outcomes, so the authors recommend a total dose of 40 Gy in five fractions as a prescription dose [52,53]. Ballas et al. performed a phase I trial in 24 patients with three dose levels: Fifteen fractions of 3.6 Gy, 10 × 4.7 Gy, and 5 × 7.1 Gy. With a median follow-up of 1.2 years, dose-escalation showed promising results, with no patients experiencing relevant severe toxicities (grade ≥ 3) [54]. A recent phase II multicenter trial in which 30–34 Gy was administered in five fractions to the prostate bed reported its dosimetric results after applying a specific bladder and rectum filling protocol. In this study, cone-beam CT intrafraction showed that CTV volume remained stable, with minimal volumetric and dosimetric changes, although patients with 95% CTV < 93% had a higher risk of CTV intrafraction volume changes [55]. In view of the recent data from the SAKK 09/10 study [56], the dose going forward in the next few years should probably be 30 Gy in five fractions as suggested by Repka et al. [51], pending validation in future clinical trials.

Detti et al. described their experience with SBRT for isolated recurrence in the PCa bed, in which 16 patients received 30 Gy in five fractions for re-irradiation or 35 Gy in five fractions for patients without prior radiotherapy treatment. With a median follow-up of 10 months, treatment tolerance was good, and only one patient experienced grade 2 acute genitourinary and gastrointestinal toxicity [57].

## 5. Contribution of Endorectal Devices in Prostate SBRT

**Figure 1:**
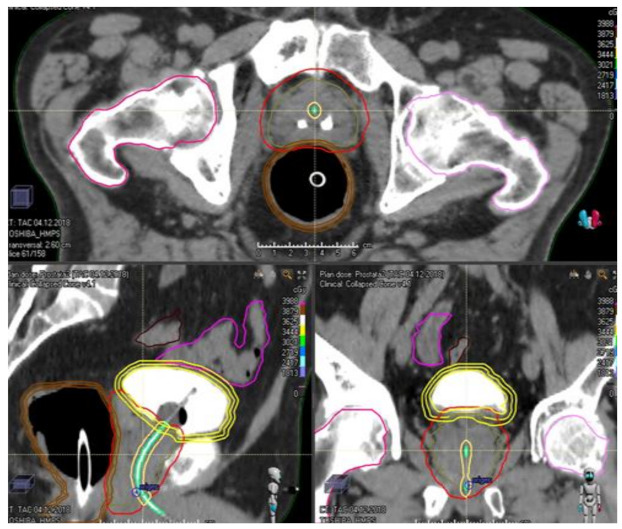
Endorectal balloon in the treatment of localized prostate cancer with SBRT.

**Figure 2:**
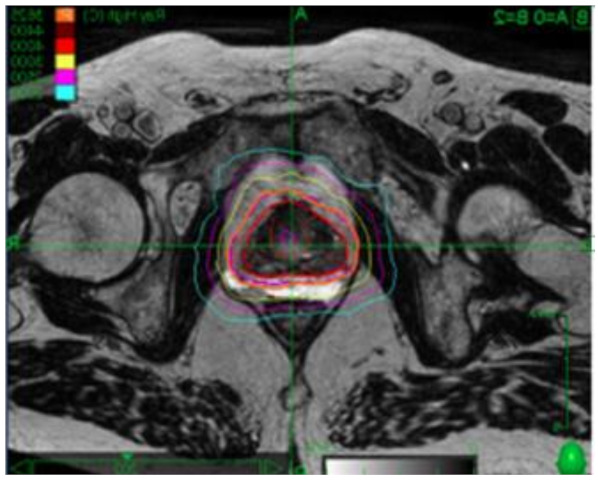
Hydrogel spacer in the treatment of localized prostate cancer with SBRT.

In SBRT treatments of PCa, it is important to reduce the variations and the movement of the organs at risk, to obtain greater precision. By using endorectal devices, it is possible to control the movement of the prostate and rectum, either by fixing the rectum or by separating it from the prostate, decreasing the exposure of the anterior-lateral rectal wall to high radiation dosage. Currently, there are different types of endorectal devices, and the most widely used are: Endorectal balloon (Figure 1), hydrogel spacer (Figure 2), and rectal retractor.

In 2017, Hamstra et al. published the results of a single-blind, randomized phase 3 clinical trial that evaluated the use of hydrogel in patients treated with IMRT. Two hundred and twenty-two patients were randomized to either placement of a hydrogel spacer or not, administering a dose of 79.2 Gy in 44 fractions to the prostate with/without seminal vesicles. The study did not meet its primary objective, which was grade 1 or greater rectal or procedural adverse events in the first six months (34.2% vs. 31.5%, *p* = 0.7), although it did show a 75% reduction in grade 1 rectal toxicity in the hydrogel spacer group (*p* < 0.03), while no grade 2 toxicity was observed in this subgroup (*p* < 0.015). Likewise, an improvement was seen in grade 1 genitourinary toxicity, along with patient-reported bowel and urinary quality of life, all favoring using the hydrogel spacer, although without differences in the dosimetric analysis of the urinary structures that would explain this improvement in urinary toxicity. It was notable the absence of any evaluation as to the reliability of patient masking in the study [58].

In 2018, the results of the dosimetric analysis of 10 patients treated with SBRT in which endorectal devices were used were published, and it enabled a reduction in the dose on the rectal wall [59], with these results later confirmed in other series [60,61,62]. More and more studies are evaluating the results of SBRT toxicity in patients in whom hydrogel spacer is used. In 2018, King et al. [63] published a small comparative study of dosimetry in treatment plans before and after the placement of hydrogel, highlighting a reduction in rectal volume that would receive 36 Gy with using hydrogel (2.6 cc on average prespacer vs. 0.3 cc postspacer, *p* = 0.031). Hwang et al. [64] published the safety and efficacy results of 50 patients treated with SBRT and hydrogel spacer with 36.25 Gy in five fractions. With a median follow-up of 1.6 years, there was no gastrointestinal toxicity or complications associated with hydrogel spacer insertion. In another prospective study of 42 patients treated for PCa with SBRT [65], a dosimetric analysis was performed comparing patients in whom hydrogel spacer was used, noting that the maximum dose received by the rectum is lower in patients in whom hydrogel spacer was used, as well as an improvement in most rectal dosimetric parameters, these results being corroborated by other studies [66,67]. A meta-analysis, published in 2020 [68], with 1011 patients, evaluated the role of hydrogel spacer in 486 patients who had hydrogel spacer inserted. The rectal separation in the hydrogel spacer group was wide, with a median distance of 11.2 mm between the rectum and prostate, while the complications related to the placement of hydrogel spacer were mild and transient, with incidence varying between 0% and 10%, and 66% less rectal irradiation described in the hydrogel spacer group at the isodosis of 70 Gy (rectum V70) compared to the controls, (*p* = 0.001). This dosimetric advantage contributed to a reduction of late grade 2 or higher rectal toxicity in patients with hydrogel spacer (1.5% vs. 5.7%; *p* = 0.05). [69]

Finally, the rectal retractor is a rigid device that reduces variations in the rectal filling, and rectal and prostate movements. Four studies show that the use of a rectal retractor can significantly reduce intrafractional movement and dose in the rectal wall. De Leon et al. [62] evaluated the intrafraction movement of the prostate and the dosimetric impact of using a rectal retractor in 10 patients who were due to have a boost of 19 Gy in two fractions after 46 Gy in 23 fractions to the pelvis, describing a reduced anterior displacement of the base and prostate apex in the sagittal plane in the rectal retractor group (*p* < 0.05), which resulted in significant improvement in rectal wall dosimetry. Rectal retractor significantly reduced rectal V16 and V14, the maximum dose, and the percentage of the posterior rectal wall that received 8.5 Gy. Nicolae et al. [70] analyzed 20 patients treated with SBRT with a dose of 26 Gy in two fractions, once a week, in which rectal retractor was used. No patient had a displacement of greater than 3 mm, and no statistically significant differences were seen between treatment plans with and without rectal retractor for the rectal and bladder contours; the authors concluded that rectal retractor allows the safe administration of extreme hypofractionation treatments, controlling the movement of the prostate, and ensuring the reproducibility of treatment.

We must consider that most of the studies published to date have not shown any clear clinical benefit to support the routine use of endorectal devices in these patients, and this adds to existing doubts about the cost-effectiveness of the use of the devices [71] or whether the oncological results might be affected in some patients by their use. We need more studies with longer to follow up [72], to define which patients could benefit particularly from using these devices.

## 6. Future Directions

Ongoing clinical trials seek to address the use of SBRT in different clinical scenarios and shed light on issues, such as total treatment dose, fractionation, concomitant use of ADT, or lymph node irradiation.

There are currently 3 phase III studies in the recruitment phase. One of them compares 36.25 Gy in five fractions with 70 Gy in 28 fractions in patients with stage IIA-B PCa, comparatively evaluating toxicity with both treatment regimens (NCT01766492). Another study compares SBRT with low-rate brachytherapy in terms of cost-effectiveness and cost-utility, as well as sexual dysfunction outcomes (NCT03830788). In the third ongoing phase III study (NCT01584258), patients for whom surgery is considered are randomized to laparoscopic prostatectomy or prostate SBRT delivered with 36.25 Gy in five fractions (PACE-A trial), and patients for whom surgery is not considered or who refuse surgery are randomized to either conventionally fractionated radiotherapy delivered to a dose of 78 Gy in two Gy fractions or SBRT delivered with 36.25 Gy in five fractions (PACE-C trial).

Regarding phase II trials, there are at least six with active recruitment, three of them in high-risk patients (Table 4), and a phase II trial, FASTR-2, completed pending publication, which evaluates the safety and efficacy of the combined use of SBRT with one year of ADT.

## 7. Conclusions

Extreme hypofractionation is a therapeutic alternative to conventional fractionation in patients with low- and intermediate-risk PCa, with a similar toxicity profile and biochemical control. SBRT data in patients with localized high-risk PCa and on the prostate bed are encouraging. However, more studies are needed to shed light on using extreme hypofractionation in these scenarios. Furthermore, well-designed studies are required to evaluate using ADT concomitant with SBRT, as well as prophylactic lymph node irradiation.

## Figures and Tables

**Table 2:** Comparison of EQD2Gy and BED figures with different radiotherapy schedules in treatment after tumor prostatectomy (α/β= 1.5 Gy) and normal late response tissues (α/β= 3 Gy).

Radiotherapy Schedules	Total Dose (Gy)	Dose/Fraction (Gy)	Number of Fractions	EQD2 (Gy_1,5_)	BED (Gy_1,5_)	EQD2 (Gy_3_)	BED (Gy_3_)
Conventional fractionation	70	2	35	70	163.3	70	116.7
Moderate hypofractionation	62.5	2.5	25	71.4	166.7	68.8	114.6
Ultra-hypofractionation–SBRT	37.25	7.25	5	90.6	211.5	74.3	123.9

EQD2, equivalent dose in 2 Gy fractions; BED, biologically effective dose.

**Table 3:** Extreme hypofractionation studies after prostatectomy.

Extreme Hypofractionation Studies after Prostatectomy	Sampath 2020			Ballas 2019	Detti 2015	
N patients	3	8	15	12	16 (50% after surgery and radiation therapy; 50% after only surgery)	
SBRT dose	7 Gy × 5, alternate days	8 Gy × 5, alternate days	9 Gy × 5, alternate days	7.1 Gy × 5, consecutive days	6 Gy × 5 after previous surgery and radiation therapy on alternate days 7 Gy × 5 after only surgery on alternate days	
ART/SRT (%)	0/100	0/100	0/100	NE	NE	
Basal PSA (medium)	0.4 ng/mL	0.4 ng/mL	0.4 ng/mL	0.05 ng/mL	Surgery + EBRT: 4.9 ng/mL Surgery: 3.3 ng/mL	
Concurrent Hormonotherapy (%)	0	50	40	33	Surgery + EBRT: 12.5% Surgery: 50%	
TOXICITY						
GU-A	G1: 33%	G1: 37.5%		G1: 40%	G1-2: 25%	G1-2: 12.5%
GI-A	G2: 33%	G1: 37.5% G2: 37.5%		G1: 33% G2: 7%	G1-2: 8%	G1-2: 12.5%
GU-T	G3: 33%	G1: 25% G2: 25% G3: 12.5%		G1: 33% G2: 27% G3: 13%	G1-2: 12.5%	0
GI-T	G2: 66%	G1: 25% G2: 12.5%		G1: 47%	0	0

EBRT, external beam radiation therapy; ART/SRT, adjuvant radiotherapy/salvage radiotherapy; PNI, perineural invasion; CK, cyberknife; CBCT, cone-beam CT; GU-A, acute genitourinary; GI-A, acute gastrointestinal; GU-L, late genitourinary; GI-L, late gastrointestinal; NS, not specified.

**Table 4:** Ongoing trials with SBRT in prostate cancer.

Identification Number NCT	Type of Study/Phase	*n*	Arm and Intervention	Primary Objective
NCT01766492	Phase III	622	Experimental arm, SBRT 36.25 Gy in five fractions. Control arm, IMRT 70 Gy in 28 fractions.	Determine whether SBRT is superior to IMRT in terms of genitourinary (GU) and gastrointestinal (GI) toxicity and quality of life.
NCT01584258	Phase III	1716	Laparoscopic prostatectomy versus SBRT for patients for whom surgery is considered. (PACE-A trial). Patients not candidates for surgery, normofractionated RT (78 Gy in 39 fractions) versus SBRT 36.25 Gy in 5 fractions (PACE-C trial)	Biochemical relapse-free survival.
NCT03830788	Phase III	240	SBRT 36.25 Gy/7.25. Gy/versus low dose rate brachytherapy	Cost-effectiveness analysis of SBRT compared to low dose rate brachytherapy, 3 years after the end of treatment.
NCT01985828	Phase II	72	Evaluates the efficacy of SBRT with CyberKnife in treating RI prostate ADC (alone and as BOOST) and AR. Experimental arm, intermediate-risk ADD (4–6 months) + CyberKnife 36.35 Gy in 5 fractions (monotherapy) ADD (4–6 months) + IMRT prostate/seminal vesicles (45–50.4 Gy) + BOOST with SBRT (21 Gy in 3 fractions). Experimental, high-risk ADD (6 months–3 years) + IMRT 45–50.4 Gy on pelvis and prostate + SBRT BOOST 21 Gy (7 Gy × 3)	Survival free of biochemical recurrence at 5 years.
NCT03294889	Phase II	45	SBRT single session 19 Gy in localized prostate cancer.	Acute GI and GU toxicity three months after the end of treatment. CTCAE v.4.03. Survival free of biochemical relapse at 3 years.
NCT01618851	Phase II	70	Evaluate the effectiveness of SBRT BOOST with Cyberknife after IMRT. IMRT 45 Gy in 25 fractions + BOOST with SBRT with Cyberknife 19.5 Gy in 3	Estimate the local relapse rate assessed by 2-year postradiotherapy prostate biopsy.
NCT03380806	Phase II	100	Compare BOOST with conventional radiotherapy vs. SBRT after pelvic radiotherapy (45 Gy in 25 fractions). In both arms, ADD is maintained for 3 years. Arm 1: Conventional pelvic radiotherapy + BOOST with conventional radiotherapy (33–35 Gy in 16 fractions). Arm 2: Conventional pelvic radiotherapy + BOOST with SBRT 19.5–21 Gy in 3 fractions.	Evaluate the quality of life at 6 months of treatment. Evaluate quality of life, late GI and GU, and IPSS toxicity at 12–24 months.
NCT02313298	Phase II	80	SBRT in localized prostate cancer. 36.25 Gy in 5 fractions.	Severe late GI and GU toxicity rate.
NCT03541850	Phase II	60	SBRT 34 Gy in 5 fractions, on alternate days, on prostate bed.	Survival free of biochemical recurrence at 5 years. Acute and late toxicity at 5 years.
